# Thermomechanical aging effects on vertical marginal gap and fracture resistance: a comparative study of Bioflx and traditional pediatric crowns

**DOI:** 10.1186/s12903-024-05053-4

**Published:** 2024-11-01

**Authors:** Asmaa Ali Emam Abo-Elsoud, Eman Mohamed Mohamady, Noha El-Sayed Fathi Abdou

**Affiliations:** 1 Pediatric Dentistry, Preventive Dentistry and Dental Public Health, Faculty of Dentistry, Suez Canal University, Ismailia city, Egypt; 2 Dental Biomaterials Department, Faculty of Dentistry, Suez Canal University, Ismailia city, Egypt; 3 Pediatric Dentistry, Preventive Dentistry and Dental Public Health, Faculty of Dentistry, Aswan University, Aswan city, Egypt

**Keywords:** Stainless steel crowns, Bioflx crowns, Zirconia crowns, Marginal gap, Fracture resistance

## Abstract

**Background:**

Various types of crowns are used for full-coverage restoration of primary teeth affected by caries, developmental defects, or after pulp therapy. Prefabricated Stainless Steel and Zirconia crowns are commonly utilized. Bioflx crowns, which blend the properties of Stainless Steel and Zirconia, provide a flexible and aesthetically pleasing alternative.

**Aim:**

This study aimed to evaluate the vertical marginal gap and fracture resistance of Bioflx pediatric crowns compared to Zirconia and Stainless Steel crowns following thermomechanical aging.

**Methods:**

This in-vitro study was conducted using mandibular second primary crowns of three different materials (*n* = 30). Crowns were divided into three groups; Zirconia crowns group (*n* = 10, Nu Smile, USA), Bioflx crowns group (*n* = 10, Nu Smile, USA) and Stainless Steel crowns group (*n* = 10, Nu Smile, USA). The crowns were cemented onto standardized acrylic resin dies and subjected to thermomechanical aging. Vertical marginal gap measurements were obtained using a USB digital microscope with an integrated camera, while fracture resistance was assessed with a universal testing machine. Data were analyzed for outliers and tested for normality using the Shapiro-Wilk or Kolmogorov-Smirnov tests, with statistical significance set at 0.05.

**Results:**

Significant differences were observed in the vertical marginal gaps among the groups after cementation and thermomechanical aging (*P* = 0.013 and *P* = 0.001, respectively). Zirconia crowns exhibited the largest average marginal gap, followed by Bioflx and Stainless Steel crowns. Stainless steel crowns demonstrated the highest fracture resistance, followed by Bioflx crowns, while Zirconia crowns showed the lowest.

**Conclusions:**

Bioflx crowns exhibit the largest vertical marginal gap but show greater fracture resistance compared to Zirconia crowns, although they are still less resistant than Stainless Steel crowns after undergoing thermomechanical aging.

## Background

The American Academy of Pediatric Dentistry recommends prefabricated full-coverage restorations for large or multisurface cavitated lesions in primary teeth [1, 2]. Stainless Steel crowns are considered the gold standard due to their cost-effectiveness, adaptability, minimal tooth reduction, and superior retention [3]. Moreover, their reliability and longevity are well-documented [4]. However, these crowns have limitations due to their metallic appearance, which is not aesthetically pleasing, and their potential for biological incompatibility. Such aesthetic concerns can adversely affect both the child’s and the parent’s perception, which cannot be disregarded [5, 6].

Consequently, aesthetic materials have been developed to replace Stainless Steel crowns, including open-faced crowns and pre-veneered stainless-steel crowns [7]. While these crowns offer an acceptable aesthetic, they present several drawbacks, such as poor gingival health, extended chair time, limited contouring or crimping ability, and the potential for veneer resin chipping or fracture [8, 9].

Zirconia crowns have been used in primary teeth as aesthetic treatment options, offering satisfactory mechanical and biological properties [10]. They are composed of polycrystalline ceramic without a glass component often referred to as “ceramic steel,” [11, 12]. The advantages of Zirconia crowns include high compressive strength, longevity, and biocompatibility [13, 14]. Their color, translucency, high polish and potential for subgingival extension minimize the risk of gingival irritation in primary teeth and ensure a natural appearance [15, 16]. However, Zirconia crowns require more extensive tooth preparation when compared to conventional Stainless Steel crowns [14, 17, 18]. Their greater hardness may cause wear on the opposing enamel [19], they can’t be easily modified or adjusted at the margins, relying solely on dental cement for retention [20]. Furthermore, their high cost also makes them more expensive compared to other aesthetic dental treatment options [21, 22].

Bioflx crowns represent a noteworthy innovation in pediatric dentistry, combining the desirable features of both Stainless Steel and Zirconia crowns. These crowns are made from a biocompatible, high-impact and high-strength hybrid resin polymer [23]. They provide an aesthetic benefit similar to Zirconia, providing a natural appearance. Bioflx crowns are highly flexibility, conforming to the anatomical cervical convexity of primary teeth and ensuring an active fit to the tooth [24]. Additionally, they require less tooth removal compared to Zirconia crowns [25, 26].

Marginal adaptation plays a crucial role in the success of full-coverage dental restorations. Achieving proper marginal adaptation ensures a tight fit between the restoration and the tooth [27]. Any discrepancies at the margins can lead to increased plaque accumulation and bacterial growth, potentially resulting in secondary caries, periodontal issues, and eventually tooth loss [1].

Another critical property that should be considered in pediatric crowns is fracture resistance which reflects both the resilience of the material and the ability to withstand the forces of chewing and other oral stresses [28]. Fracture resistance is measured by the material’s capacity to inhibit the progression of cracks that begin from inherent flaws, potentially leading to minor fractures at the restoration’s edges or extensive fractures within the filling itself [29].

Finding aesthetically pleasing and functional coverage solutions for primary molars is a key concern for pediatric dentists. Consequently, there is an immediate need to identify new materials that are cost-effective, repairable, and tailored to specific requirements. Ideally, such materials would support less invasive preparations, enhance fit and retention, shorten chair time, and simplify the placement process. Therefore, this study aims to compare the recently developed Bioflx crowns with two traditional pediatric crowns, Zirconia and Stainless Steel, in terms of vertical marginal gap and fracture resistance.

The null hypothesis of this study asserted that there were no notable differences in the vertical marginal gap and fracture resistance between Zirconia, Bioflx, and Stainless Steel crowns after undergoing thermomechanical aging.

## Methods

This in-vitro experiment was carried out in the Dental Biomaterials Department at the Faculty of Dentistry, Suez Canal University, with the approval of the institution’s Research Ethical Committee (approval number 754/2023).

Sample size calculation was performed using G*Power version 3.1.9.2 [30], based on the effect size of 0.80 using an alpha (α) level of 0.05 and Beta (β) level of 0.20, corresponding to a power of 80%. Consequently, the calculated minimum number of samples required was thirty.

### Grouping

**Fig. 1:**
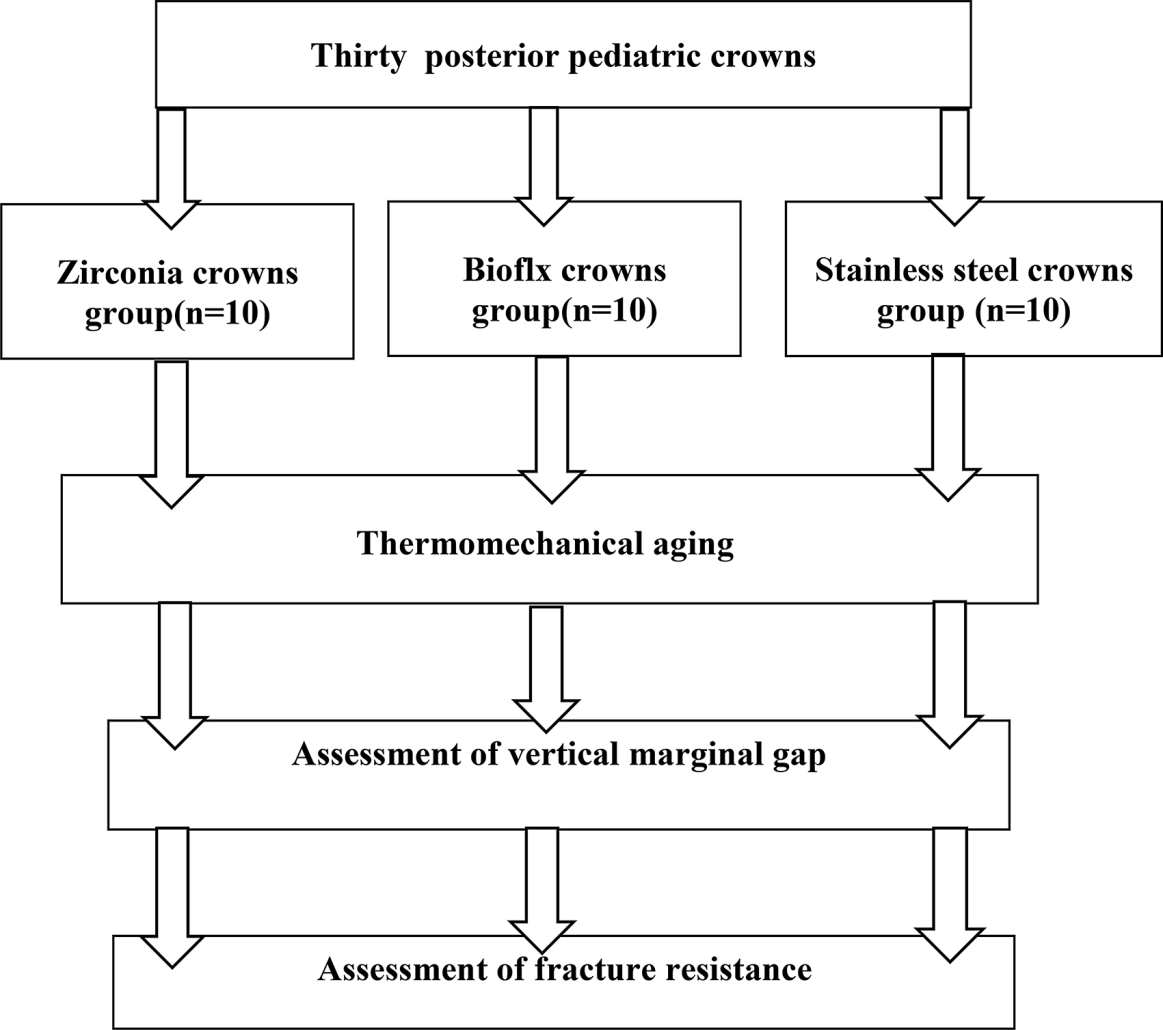
Schematic representation of experimental study design

Thirty-three primary mandibular right second molar crowns size five were used in this study (*n* = 33) (3 crowns used for die fabrication were excluded from the laboratory tests). The crowns (*n* = 30) were equally divided into three groups; Zirconia crowns group (*n* = 10, Nu Smile, USA), Bioflx crowns group (*n* = 10, Nu Smile, USA) and Stainless Steel crowns group (*n* = 10, Nu Smile, USA). (Fig. 1)

### Die fabrication

Initially, three acrylic dies were produced by filling one crown of each type with acrylic resin (Cold cure acrylic resin, Acrostone, Egypt) and allowing them to harden for one hour. The dies were then evaluated for fit, and any visible undercuts in the dies were removed with a finishing bur. The dies were placed in a PVC (polyvinyl chloride) tube, filled with the same acrylic resin to form a die resin base and left to solidify for an additional hour. Impressions of each crown die were taken using silicone impression material (Zhermack S.p.A Tropicalgin, Italy), which hardened within an hour. This process yielded three negative master impressions, which were then used to precisely fabricate ten acrylic dies for each crown category, subsequently the dies cured for 24 h [15, 31].

### Crowns cementation

**Fig. 2:**
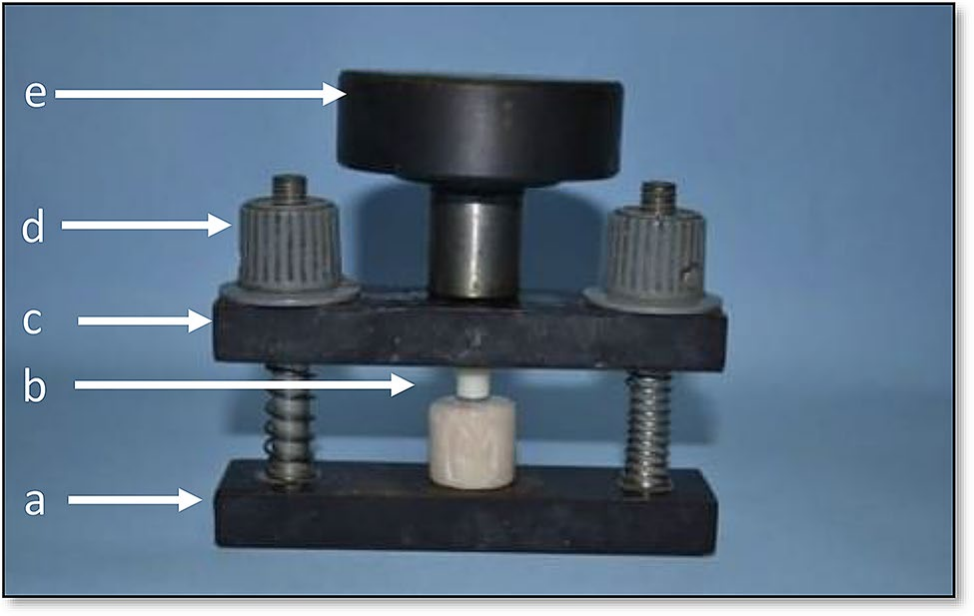
Cementation holding device. (**a**) Fixed base portion. (**b**) Position of a crown sample (**c**) Upper movable portion. (**d**) Tightening plastic cups. (**e**) T shape weight-bearing portion (3 kg)

Thirty crowns were cemented onto acrylic dies using glass ionomer cement (Riva self-cure, SDI, Australia), prepared according to the manufacturer’s instructions and applied to the inner surfaces of the crowns. The crowns were then accurately positioned on the relevant dies and allowed to harden for 10 min under static finger pressure, followed by axially loaded with a 3 kg weight using a custom-made holding device [32] (Fig. 2). Excess cement was carefully removed with a dental explorer. Finally, the crowns were left to be completely set for 24 h [33].

### Thermomechanical aging

To replicate the conditions of six months in the oral environment, the crowns underwent 5000 thermal cycles ranging from 5 to 55 °C, with a dwell time of 25 s and a lag time of 10 s using a Robota automated thermal cycler (BILGE, Turkey) [34]. Subsequently, a chewing simulator (Robota, model ACH-09075DCT, AD-Tech Technology CO., LTD., Germany) was employed to administer 75,000 cycles of a 50 N occlusal load at a frequency of 1.6 Hz [28, 35].

### Assessment methods

The vertical marginal gap of the thirty crowns was assessed first, followed by an evaluation of their fracture resistance. The entire procedure was performed by a single operator. The validity and reliability were assessed as the intra-observer reliability for the measurements.

### Assessment of vertical marginal gap

Marginal discrepancy was assessed by measuring the vertical distance between both the crown and corresponding die margins (parallel to the tooth axis). Each sample was documented using a USB digital microscope equipped with an integrated camera; the photographs were captured using the specified image acquisition system [27, 36–39]. A digital camera (U500x Digital Microscope, Guangdong, China) with a resolution of 3 megapixels were positioned vertically 2.5 cm away from the samples. The images were captured at the highest resolution and interfaced with a compatible personal computer set to a fixed magnification of 40X. Gap width measurements were conducted using a digital image analysis system (Image J 1.43U, National Institute of Health, USA). Calibration was made by comparing an object of known size (a ruler in this study) with a scale generated by the Image J software. Shots of the margins were taken for each specimen for all surfaces (buccal, mesial, lingual and distal). Finally, morphometric measurements were done for each shot (4 equidistant landmarks along the circumference for each surface) [40]. The average values, along with standard deviations, were expressed in micrometres (µm).

### Assessment of fracture resistance

After thermomechanical aging, each sample was positioned on a computer-controlled materials testing apparatus (Model 3345; Instron Industrial Products, Norwood, MA, USA) that featured a 5 kN load cell following ISO specifications No ISO 7500-1. Data collection was conducted using Bluehill Lite Software (Instron^®^). The samples were firmly attached to the lower fixed part of the machine using screws. Fracture testing proceeded in compressive mode, with the force directed occlusal via a metallic rod with a rounded tip (5.6 mm diameter) connected to the upper movable part of the machine. The machine functioned at a crosshead speed of 1 mm/min, and a tin foil sheet was inserted between the specimen and the rod to facilitate uniform stress distribution and minimize localized force spikes. The occurrence of a fracture was signalled by an audible crack and a noticeable decline in the load-deflection curve which was recorded by Software. The force required to induce the fracture was quantified in Newtons (N) [14, 16, 28]. The Mode of failure of the tested crowns was assessed using a light microscope (Leica Microsystems GmbH, Germany) to detect and verify the surface deformation, perforations and fractures [41].

### Statistical analysis

Data compilation, verification, and organization into tables were performed using Microsoft Excel 2016. The dataset underwent outlier detection and normality testing, using the Shapiro-Wilk and Kolmogorov-Smirnov tests at a 0.05 significance level. Parametric descriptive statistics were presented numerically in tables, with data represented as mean and standard deviation. For inferential statistics, a one-way Analysis of Variance (ANOVA) was performed, followed by Duncan’s Multiple Range Test (DMRT) at 0.05 level. Statistical analyses were performed using IBM-SPSS version 29.0 form Mac OS (IBM SPSS, release 2023, Armonk, NY: IBM Corp).

## Results

The results in Table 1 showed a significant difference in the vertical marginal gap between groups (Zirconia, Bioflx, and Stainless Steel) across all surfaces before cementation, after cementation, and after aging, except at the distal surface after cementation and after aging.

**Fig. 3:**
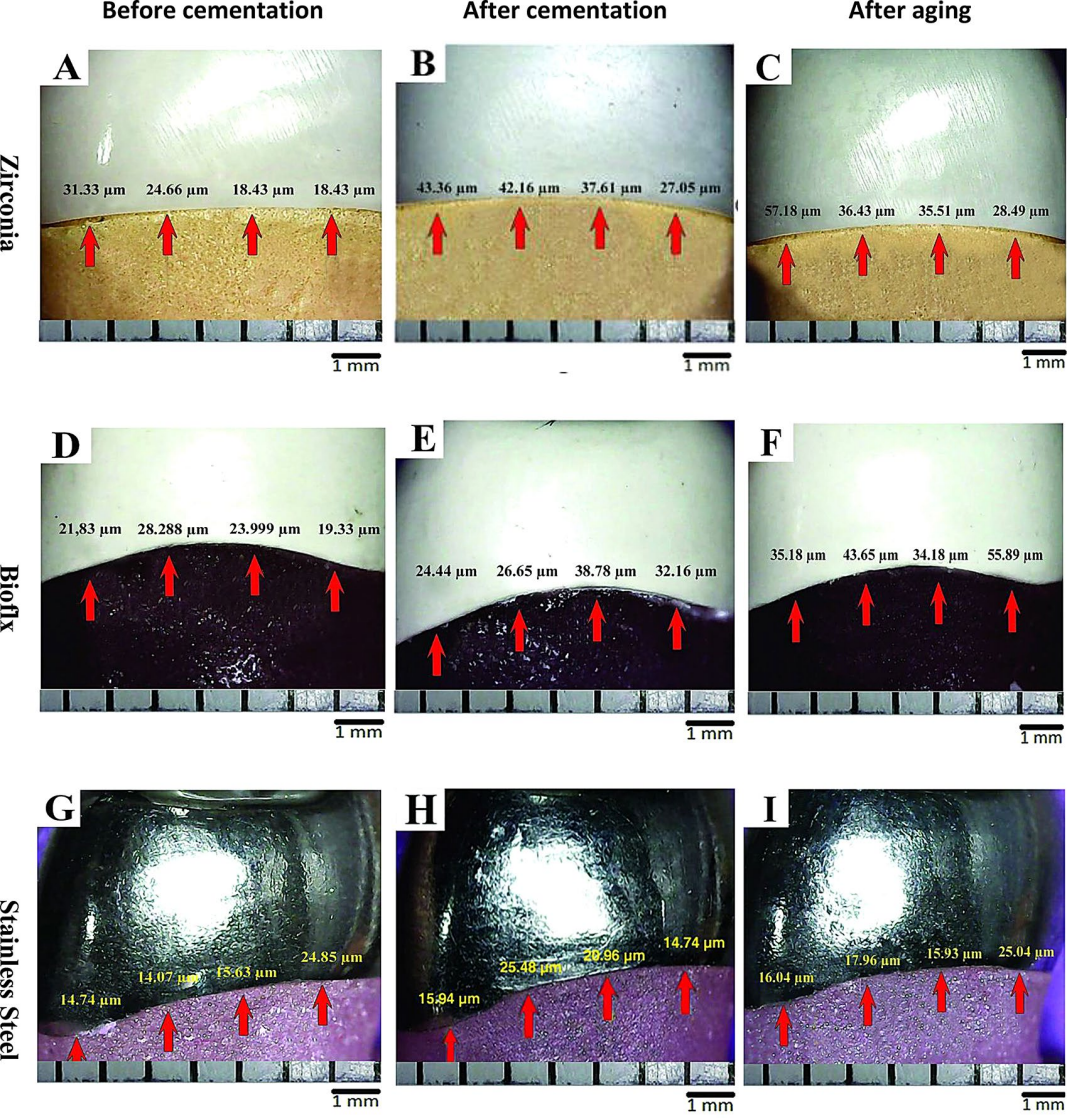
Digital light microscope images showing the vertical marginal gap of the tested crowns. Zirconia crowns (**A**; before cementation, **B**; after cementation and **C**; after thermomechanical aging), Bioflx crowns (**D**; before cementation, **E**; after cementation, and **F**; after thermomechanical aging) and Stainless Steel crowns (**G**; before cementation, **H**; after cementation, and **I**; after thermomechanical aging). Arrows refer to equidistant landmarks along the circumference for each surface

Statistical analysis showed no significant differences in the average of vertical marginal gap between Zirconia, Bioflx, and Stainless Steel crowns before cementation (*P* = 0.063), while after cementation and after aging showed significant differences between groups (*P* = 0.013 and 0.001 respectively). The pairwise comparison revealed a statistically significant difference in the percentage change of vertical marginal gap among groups after cementation, with Zirconia showing a 39.3% change, Bioflex showing a 40.2% change, and Stainless Steel showing a higher change of 58.9%. Additionally, after aging, there was a significant difference in the percentage change where Zirconia exhibited a 93.8% change, Bioflex a 117.0% change, and Stainless Steel a lower change of 75.4%. (Table 2; Fig. 3).

**Table 1:** Mean and standard deviation SD (±) values (µm) of the vertical marginal gap among different groups across all surfaces before cementation, after cementation and after thermomechanical aging

		Zirconia	Bioflx	Stainless Steel	F test	*P* value
		Mean ± SD	Mean ± SD	Mean ± SD		
Buccal	Before cementation	31.14 ± 1.64^b^	26.86 ± 4.29^c^	35.08 ± 5.28^a^	5.178	0.024**
	After cementation	45.08 ± 2.51^a^	31.62 ± 7.15^c^	42.38 ± 3.26^b^	11.173	0.002**
	After aging	53.86 ± 2.45^a^	40.38 ± 7.52^c^	47.58 ± 4.^87b^	7.918	0.006**
Mesial	Before cementation	35.70 ± 2.33^a^	27.72 ± 4.38^b^	27.66 ± 8.00^b^	1.162	0.034**
	After cementationt	47.14 ± 1.55^b^	50.38 ± 4.72^a^	44.82 ± 7.48^b^	0.426	0.0461**
	After aging	74.64 ± 10.23^a^	74.28 ± 7.44^a^	49.12 ± 5.67^b^	7.281	0.009**
Lingual	Before cementation	31.94 ± 1.87^a^	27.52 ± 8.06^b^	28.40 ± 4.49^b^	0.927	0.0422**
	After cementationt	44.42 ± 4.13^a^	31.86 ± 6.44^b^	46.84 ± 5.52^a^	10.901	0.002**
	After aging	57.20 ± 6.49^b^	63.32 ± 2.58^a^	46.54 ± 5.19^c^	14.272	0.001**
Distal	Before cementation	30.50 ± 2.76^a^	26.28 ± 5.41^b^	22.72 ± 2.30^c^	5.387	0.021**
	After cementationt	43.54 ± 2.78^b^	38.26 ± 7.10^a^	47.12 ± 8.84^a^	2.188	0.155
	After aging	64.60 ± 4.15^a^	57.32 ± 7.96^a^	54.74 ± 6.32^a^	3.255	0.074

** Means significant at P <0.05

Different superscript letters indicate statistically significant differences among groups at P<0.05

**Table 2:** Mean, standard deviation SD (±) values (µm) and percentage change of the vertical marginal gap among different groups before cementation, after cementation, and after thermomechanical aging

	Before cementation (A)	After cementation (B)	After aging (C)	% change (B-A)	% change (A-C)	F test	*P* value
Zirconia crowns	32.3 ± 1.0 ^c A^	45.0 ± 0.8 ^b A^	62.6 ± 2.6 ^a A^	**39.3**	**93.8**	424.4	< 0.001**
Bioflx crowns	27.1 ± 4.8 ^c A^	38.0 ± 5.3 ^b B^	58.8 ± 6.5 ^a A^	**40.2**	**117.0**	41.83	< 0.001**
Stainless Steel crowns	28.5 ± 2.7 ^c A^	45.3 ± 3.4 ^b A^	50.0 ± 1.6 ^a B^	**58.9**	**75.4**	90.79	< 0.001**
F test	3.51	6.36	12.19				
P value	0.063	0.013**	0.001**				

**; Means significant differences P<0.05

Small superscript letters (Row) mean the significant difference between before cementation, after cementation and after aging at P<0.05

Capital superscript letters (Column) mean significant difference among groups at P<0.05

**Fig. 4:**
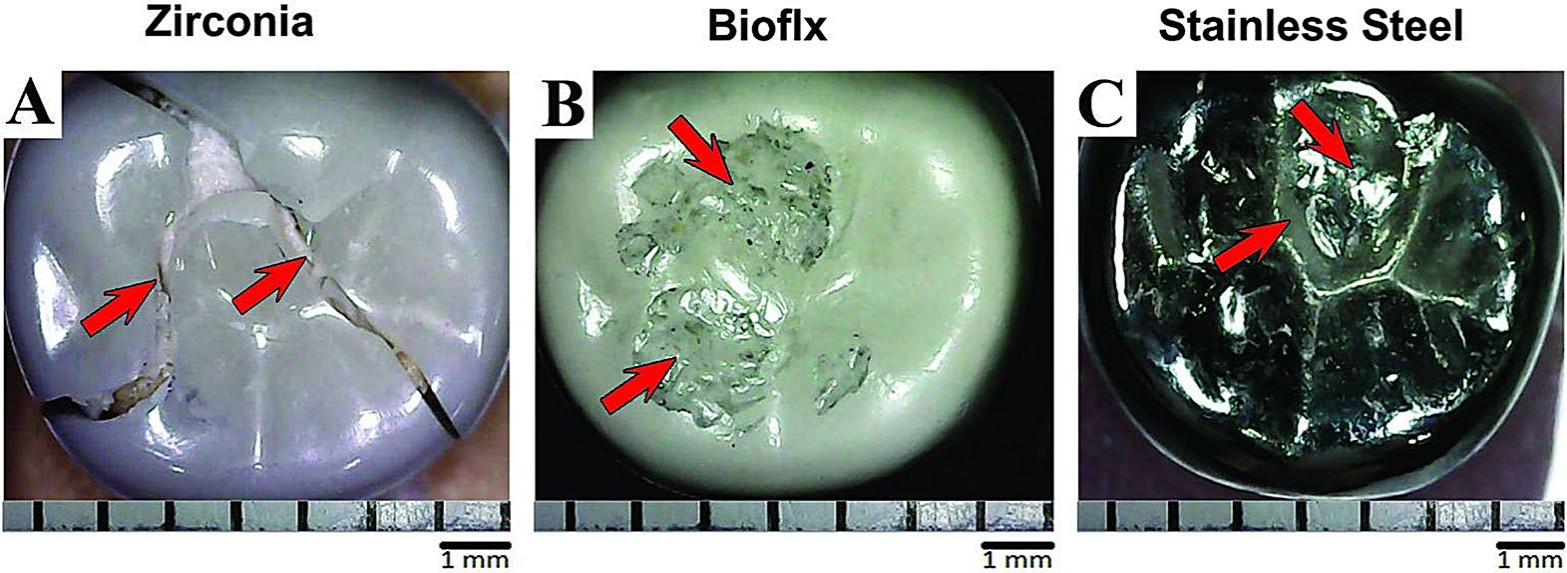
Light microscope images showing the mode of failure among tested crowns after thermomechanical aging. (**A**) Arrows refer to the fracture line in Zirconia crowns. (**B**) Arrows refer to the permanent surface deformation in Bioflx crowns. (**C**) Arrows refer to permanent surface deformation in Stainless Steel crowns

The fracture resistance test results after thermomechanical aging revealed a significant difference among the study groups (*P* < 0.001). Stainless steel crowns exhibited the highest mean fracture resistance (3062.14 ± 408.97) followed by Bioflx crowns (2403.44 ± 92.65), while Zirconia crowns demonstrated the lowest resistance (1286.30 ± 91.56) among different study groups, Table 3. The failure mode for Stainless Steel and Bioflx crowns was characterized by permanent deformation of the occlusal surface and micro perforations, whereas Zirconia crowns experienced fracture lines (Table 4; Fig. 4).

**Table 3:** Mean and standard deviation SD (±) values (N) of fracture loads among groups after thermomechanical aging

	Mean ± SD	F test	*P* value
Zirconia crowns	1286.30 ^c^ ± 91.56	65.619	< 0.001**
Bioflex crowns	2403.44 ^b^ ± 92.65		
Stainless Steel crowns	3062.14 ^a^ ± 408.97		

**; Means significant differences

Small letters mean the significant difference among groups at P<0.05

**Table 4:** Quantitative analysis of the failure mode among groups

Crown type	Surface deformation	Micro perforations	Fractures and loss
Zirconia crowns	----	-----	10
Bioflx crowns	10	4	----
Stainless Steel crowns	10	6	----

## Discussion

With advancements in aesthetic dentistry, the demand for more visually appealing alternatives to traditional Stainless Steel crowns is increasing among both young patients and their guardians [24, 25].

While utilizing natural teeth in in-vitro studies can closely mimic actual clinical conditions, this study used resin dies to avoid the inherent variability of natural teeth. Differences in tooth anatomy, hard tissue thickness, age, storage conditions, shape, and size complicate the standardization of tooth preparation. Such variability could lead to inconsistent cement thickness across samples, increasing stress on the restorative material and raising the risk of fractures [42].

Thermal and mechanical cycling was used to simulate the aging of restorations and replicate the impact of masticatory forces on the crowns as if they had been in the oral cavity for six months [43]. Cyclic loading mimics the repetitive sub-threshold stresses from daily chewing activities, which can contribute to the failure of restorations. In this study, thermomechanical aging provides a more accurate assessment of the crowns’ performance and offers insights into their potential clinical lifespan [15, 44].

In this study, the assessment of marginal gaps was performed using a USB digital microscope with integrated illumination. This non-destructive method is preferred as it is external and does not require intermediate materials like impression materials. It is also less time-consuming than other techniques and minimizes potential errors from multiple steps, improving result accuracy. Furthermore, this method is cost-effective. The evaluation focused on the vertical cervical marginal gap, a commonly used indicator of restoration fit precision [27, 36–39].

The averaged vertical marginal gap values for the buccal, mesial, lingual and distal surfaces were reported for simplicity, clarity, and ease of comparison with other studies. The findings revealed no significant differences in the marginal gaps among the Zirconia, Bioflx, and Stainless-Steel crowns before cementation. However, significant differences were observed among groups after cementation and following aging.

It has been established that a maximum marginal discrepancy of 120 μm is deemed clinically acceptable for traditionally manufactured restorations [39, 45]. The average discrepancy values for all crowns examined at various stages in this study remained consistently below or within this acceptable range, indicating that the fit of all crowns is clinically suitable [46, 47].

This study’s findings align with those of several researchers [48–50] who noted a considerable increase in the marginal gap post-cementation compared to pre-cementation. Variations in cementation techniques, such as unregulated manual pressure (thus, a standardized pressure was applied using a specialized device in this study) or the over-application of cement, can lead to inconsistent cement distribution, resulting in one side of the axial wall having a thicker cement layer while the opposite side has a thinner one [51, 52].

In this study, there was a significant increase in marginal discrepancy values among groups following thermomechanical aging. These findings are consistent with those reported by Fouad, M. [36], who indicated that thermal aging could exacerbate marginal discrepancies between crowns and their corresponding dies. This phenomenon may arise from the cementation process, which is influenced by various factors, including the thermal mismatch between the dies and the crowns. Furthermore, Al-Haj Ali et al. [53], and Elsayed et al. [54] found that Zirconia crowns exhibited the most significant microleakage, potentially linked to artificial aging and thermocycling. These processes are crucial as they tend to diminish the adhesive strength between Zirconia crowns and luting cements. Additionally, thermocycling can cause repeated stresses from shrinkage and expansion, result from the mismatching of thermal coefficients of the materials used in the restoration [55].

In fracture resistance measurements, every tested specimen withstood a load greater than the typical occlusal forces observed in the posterior region of children’s mouths during the transition from early primary to permanent dentition. According to research by Talekar et al. [56], the average occlusal biting forces were recorded as 176 N in early primary dentition, 240 N in late primary dentition, 289 N in early mixed dentition, and 433 N in late mixed dentition.

In this study, the findings of fracture resistance among groups after thermomechanical aging noted a distinction among Zirconia, Bioflx, and Stainless Steel crowns. Stainless Steel crowns exhibited the greatest average fracture resistance, followed by Bioflx crowns, while Zirconia crowns displayed the lowest fracture resistance.

The findings on fracture resistance from this study align with the findings reported by Kist et al. [13] and Elsayed et al. [54] who investigated the fracture load and chewing simulation of Zirconia and Stainless-Steel crowns for primary molars. Their research documented the high fracture load values for Stainless-Steel crowns that might be a result of the higher ductility of the Stainless-Steel crowns compared with the more brittle Zirconia. Additionally, Townsend et al. [57], and Akila et al. [1], reported that Stainless Steel crowns exhibited better compressive strength compared to Zirconia crowns.

The primary types of damage observed in Stainless Steel crowns are fatigue and permanent occlusal surface deformation which is considered a functional failure. Fatigue damage occurs because metals, (Stainless Steel), are susceptible to weakening under repeated alternating loads. This susceptibility is compounded by potential thinning of the occlusal surface due to plastic deformation from chewing simulations. Over time, the repeated stresses can lead to noticeable deformations in the crowns [58]. Despite their durability as restorative materials for deciduous teeth, Stainless Steel crowns can suffer damage and deformation over long-term use if subjected to forces exceeding typical chewing pressures [59].

Zirconia crowns showed the lowest fracture resistance and presented with cracks and fracture lines on the occlusal surface after being subjected to compressive loading which is considered a brittle fracture. The vulnerability of these crowns to fracture can be attributed to their inability to absorb significant amounts of plastic strain energy [60]. When subjected to excessive force, they are likely to break, as is typical with ceramic materials, these crowns do not withstand flexure well and are prone to easy fracturing. Unlike other materials (Stainless Steel), Zirconia crowns don’t flex and can’t withstand permanent deformation and is susceptible to fracture under excessive compressive load [61, 62].

The average fracture resistance for Bioflx crowns ranks them slightly less than Stainless Steel crowns yet more than Zirconia crowns. The observed occlusal failures are characterized by fatigue and permanent occlusal surface deformation which may be attributed to the crowns’ composition of a biocompatible hybrid resin polymer, which possesses more flexibility and greater elasticity. This material is designed to improve ductility, adaptability, and durability [25, 26].

This finding aligns with Deolikar et al. [26], who concluded that the Bioflx crown can withstand perpendicular forces and has superior resistance to crown fracture compared to zirconia crowns.

The null hypothesis was rejected, as significant differences were observed in both marginal integrity and fracture resistance among the Zirconia, Bioflx, and Stainless Steel crowns following thermomechanical aging.

Bioflx crowns have emerged as a promising new flexible and aesthetic alternative to both Zirconia and Stainless Steel crowns in pediatric dentistry.

This study encounters several limitations, as this study considers pioneering for investigation of Bioflx crowns and calculating the percentage change of a marginal gap of the tested groups however, limited literature was available. In addition, this study assesses 2D measurements of the vertical cervical marginal gap rather than the 3D space of the margin (depth as well as height).

## Conclusions

After thermomechanical aging, Bioflx crowns exhibit the largest vertical marginal gap when compared to Zirconia and Stainless Steel crowns. In terms of fracture resistance Bioflx crowns are greater than Zirconia crowns, while the Stainless Steel crowns offer the highest fracture resistance among the three groups.

The failure mode for Stainless Steel and Bioflx crowns is characterized by permanent deformation of the occlusal surface and micro perforations, whereas Zirconia crowns experience fracture lines.

### Recommendations

Further studies are needed to evaluate the physical and mechanical properties of Bioflx crowns, both in vitro and in vivo studies. Furthermore, a 3D assessment provides a more comprehensive evaluation of the crown’s fit.

## Data Availability

Data cannot be shared openly but are available on request from authors.
